# Patient-centred anti-platelet therapy following coronary artery bypass graft surgery

**DOI:** 10.1007/s11239-026-03331-2

**Published:** 2026-05-29

**Authors:** Eleonora De Laurentis, Nikhil Sahdev, Marjan Jahangiri

**Affiliations:** 1 Department of Cardiac Surgery, St. George’s Hospital, London, UK; 2 Department of Cardiothoracic Surgery, St. George’s Healthcare NHS Trust, London, UK

**Keywords:** Antiplatelet, Thrombosis, Coronary artery bypass graft surgery

## Abstract

While lifelong aspirin mono-therapy remains the standard of care following coronary artery bypass graft surgery (CABG), the optimal antiplatelet strategy remains a subject of ongoing debate. The evidence argues against a ‘one-size-fits-all’ strategy and supports a patient-centred approach. Antiplatelet treatment decisions should incorporate bleeding risk which is often multifactorial and necessitates careful individualised evaluation based on patient characteristics and procedural factors. Conduit selection should also be considered as graft failure following CABG remains an important problem and varies according to conduit type, highlighting the critical role of antiplatelet therapy. Saphenous vein grafts are particularly susceptible to early thrombosis and late atherosclerotic degeneration, making optimisation of antiplatelet therapy especially relevant for preserving vein graft patency, whereas its impact on arterial grafts is less well defined. Current guideline recommendations are largely extrapolated from studies involving patients with acute or chronic coronary syndromes treated with percutaneous intervention or medical therapy. Clinical presentation is also a key determinant of antiplatelet strategy. Although current guidelines support resumption of dual antiplatelet therapy (DAPT) after CABG in patients with acute coronary syndromes (ACS), these recommendations are largely inherited from CABG subgroups of broader ACS trials rather than dedicated CABG randomised studies, and recent CABG-specific evidence has challenged the routine use of ticagrelor-based DAPT in this setting. DAPT in chronic coronary syndromes may be considered only in selected patients at low bleeding risk. Although, observational and randomised data suggest that DAPT can improve vein graft patency, but this has not consistently translated into short-term clinical benefit and is offset by increased bleeding risk. In fact, emerging evidence suggests shorter or de-escalated DAPT strategies may offer a more favourable balance between graft protection and bleeding.

**Graphical Abstract:**

Patient-centred antiplatelet therapy post CABG. CABG – coronary artery bypass graft surgery; ACS – acute coronary syndrome; CCS – chronic coronary syndrome; SAPT – single anti-platelet therapy; DAPT – dual anti-platelet therapy.

**Figure:**
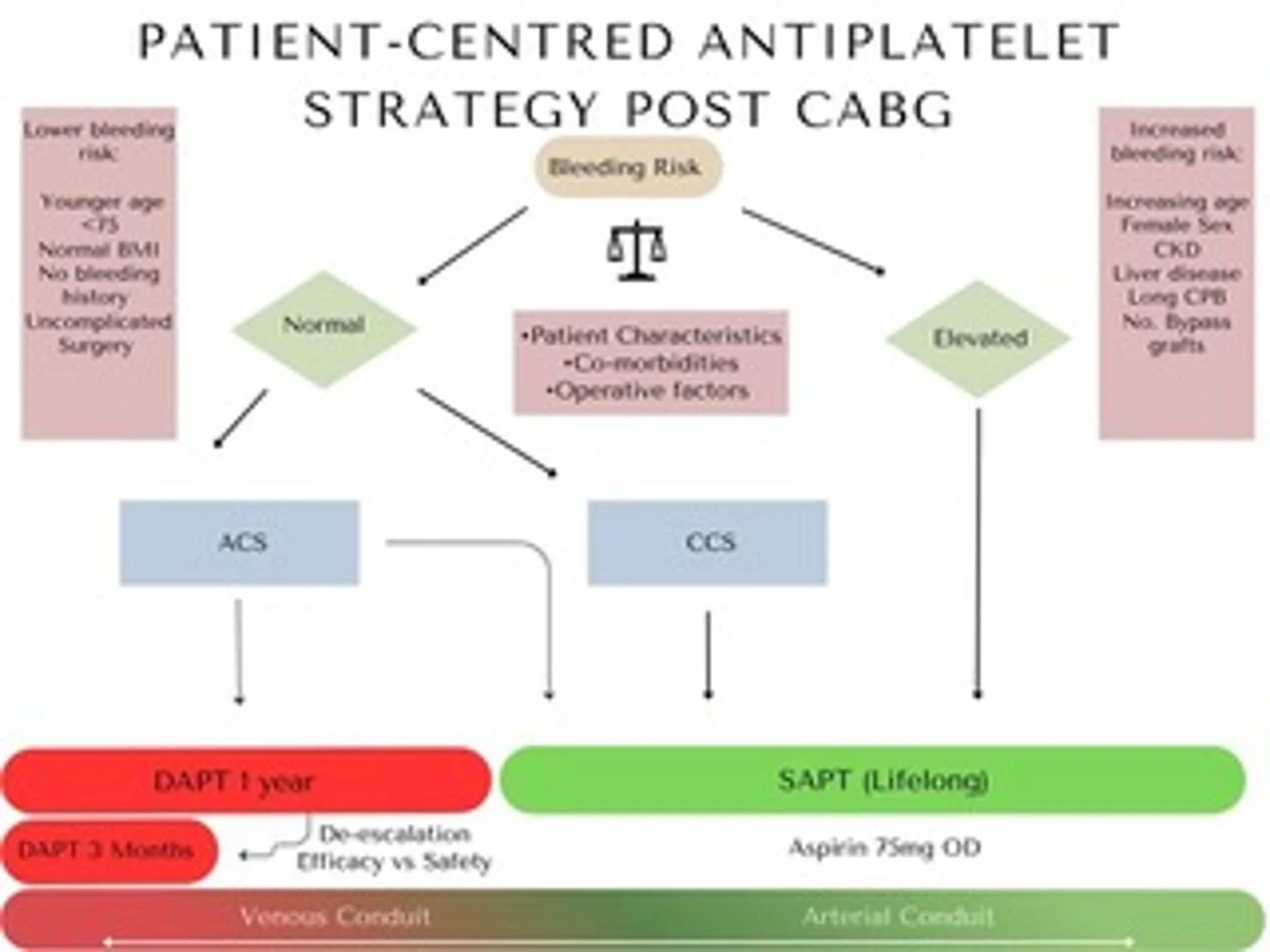

## Introduction

Antiplatelet therapy is a key element of primary and secondary prevention in coronary artery disease (CAD). Patients undergoing coronary artery bypass graft (CABG) surgery require lifelong antiplatelet therapy to prevent the risk of graft failure and reduce the risk of cardiovascular events [1], as the clinical benefit of CABG depends on long-term graft patency. The patency of bypass grafts protects against myocardial infarction (MI) and reduces the need for repeat revascularisation [2].

The optimal antiplatelet strategy following CABG remains uncertain. Most patients undergoing CABG are treated with single antiplatelet therapy (SAPT) using aspirin. However, selected patients receive dual antiplatelet therapy (DAPT) consisting of aspirin plus an oral P2Y12 receptor inhibitor, particularly when thrombotic risk is high, such as in the setting of recent percutaneous coronary intervention (PCI) or acute coronary syndrome (ACS). Some patients receive combined antithrombotic regimens, consisting of SAPT or DAPT together with oral anticoagulation (OAC), particularly those with atrial fibrillation [3, 4].

The problem with antithrombotic therapies following CABG is that it requires a patient-centred balance of ischaemic and bleeding risks. Indeed, while antithrombotic therapy reduces ischaemic risk, it simultaneously increases bleeding risk, depending on patient characteristics, the type of treatment, and its duration [3].

Only a few randomised clinical trials have been specifically conducted in the CABG population; therefore, current guideline recommendations are largely extrapolated from studies involving patients with acute or chronic coronary syndromes treated with PCI or medical therapy. However, it is important to recognise that post-CABG physiology differs fundamentally from that following PCI. PCI is primarily directed at treating flow-limiting lesions, whereas CABG provides blood flow distal to lesion and therefore protects against future lesions [5].

This article is a consensus based on the current evidence and guideline recommendations regarding antiplatelet therapy after CABG.

## Graft patency and failure rates

Achieving long-term graft patency is fundamental after CABG; however, graft failure still occurs and represents an important clinical concern as it is associated with increased risk of MI and repeat revascularisation [6]. Graft failure rates vary according to the type of conduit used in CABG.

Saphenous vein grafts (SVG) demonstrate a one-year patency rate ranging from 81% to 98% [7]. Midterm SVG patency, assessed between 5 and 7 years, declines to 75%–86% [8], while late patency at ≥ 10 years is reported at 55%–60% [9]. In these studies, the majority of patients were studied prior to the widespread use of statins and more potent antiplatelets; more recent studies suggest a patency rate of around 70% [10]. The timing of saphenous vein graft (SVG) failure across early (0–30 days), intermediate (1–12 months), and late (> 1 year) postoperative phases provides insight into the underlying mechanisms of failure [11]. Early graft failure (hours to < one month) is commonly driven by acute thrombosis secondary to damage to the endothelial and smooth muscle cells. Between one month and one year after CABG, the predominant mechanism of failure is intimal hyperplasia. Beyond 12 months, SVG failure is more commonly driven by atherosclerotic changes, including foam cell accumulation, necrotic core formation, and cholesterol deposition.

In contrast, arterial grafts show superior long-term durability. The left internal thoracic artery (LITA) demonstrates patency rates of 93%–96% at one year, 88%–94% at 5 years, and 85%–90% at ≥ 10 years [9]. Similarly, the radial artery (RA) exhibits high patency, with rates of 89%–92% at one year [8], 90%–98% at 5 years, and 89%–91% at 10 years, as reported in the RAPCO trial [12]. Failure of arterial grafts usually arises from technical issues and competitive flow or spasm in the early period, while late failure is primarily driven by slow atherosclerotic progression of native coronary artery disease (CAD).

However, in recent years the patency of coronary bypass grafts has improved significantly. The fact that half of coronary bypass grafts fail within 10 years is based on historical data. Recent reports support far better graft performance, with patency rates of 90% for LITA, 85% for radial arteries, and 70% for venous grafts at a median follow-up of 8.6 years following CABG [10].

Given the differences in patency of various grafts used in patients undergoing CABG, antiplatelet and antithrombotic strategies may have differential effects depending on graft type. SVGs are particularly susceptible to early thrombosis and late atherosclerotic degeneration, making graft patency highly dependent on effective platelet inhibition. In contrast, arterial grafts demonstrate superior intrinsic resistance to thrombosis and atherosclerosis, which may partly explain their sustained long-term patency. Consequently, optimisation of antiplatelet therapy is especially critical for preserving SVG patency, while its relative impact on arterial grafts is not well characterised [13].

## Commonly used antiplatelets

The primary antiplatelet agents used in CAD include aspirin, clopidogrel, ticagrelor, and prasugrel.

Aspirin irreversibly inhibits platelet cyclooxygenase-1 (COX-1) by acetylating a serine residue near its active site, thereby suppressing thromboxane A₂ production and platelet aggregation [14]. Aspirin remains the cornerstone of antiplatelet therapy after CABG and is recommended lifelong after CABG [15, 16].

P2Y12 receptor inhibitors represent the second major class of antiplatelet agents. Clopidogrel and prasugrel are thienopyridine prodrugs that irreversibly inhibit the platelet P2Y12 receptor after hepatic activation, whereas ticagrelor is a direct-acting, reversible P2Y12 inhibitor and has a faster onset of action [17]. Prasugrel and ticagrelor provide more potent platelet inhibition than clopidogrel but are associated with higher bleeding risk [18].

Balancing the antiplatelet potency of P2Y12 inhibitors against the risk of bleeding remains a key clinical challenge. In a retrospective analysis of the TRITON–TIMI 38 CABG cohort, prasugrel was associated with increased perioperative bleeding but significantly lower 30-day and long-term mortality (median follow up 15 months) compared with clopidogrel in ACS patients undergoing CABG, suggesting a potential survival benefit despite greater bleeding risk [19]. Similarly, in the CABG subgroup of the PLATO trial, ticagrelor in combination with aspirin significantly reduced both total and cardiovascular mortality compared with clopidogrel plus aspirin (12 months follow up), without an increase in CABG-related bleeding supporting more potent DAPT [20]. These mortality benefits were observed over relatively short to medium term follow-up (12–15 months), demonstrating the early impact of more potent P2Y12 inhibition (ticagrelor and prasugrel) in ACS patients undergoing CABG. However, it should be noted that neither trial was specifically designed to evaluate postoperative antiplatelet strategies in CABGpatients.

## Assessment of bleeding risk

In patients undergoing CABG, the risk of bleeding is substantially higher then after PCI and the clinical consequences are more catastrophic. CABG involves heparinisation, multiple anastomoses and cardiopulmonary bypass induced coagulopathy. Therefore, early post-operative bleeding has the potential to result in tamponade, re-exploration, infection and increased mortality. Bleeding also has longer term consequences such as impacts on quality of life [21] and long-term survival [22].

Surgical bleeding is most common within the first 24 h after surgery, during which platelet dysfunction related to cardiopulmonary bypass is most pronounced and begins to normalise thereafter [23]. Therefore, initiation of a second antiplatelet agent within the first 24 h is generally discouraged. Beyond this period, bleeding risk should be reassessed based on patient characteristics, comorbidities and procedural aspects (Table 1) [24, 25]. When bleeding risk exceeds a critical threshold, addition of a second antiplatelet agent should be avoided, even in patients with high ischaemic risk, as the harm associated with bleeding in CABG outweighs any potential benefit later.

**Table 1:** Factors affecting bleeding in patients undergoing CABG

Patient Characteristics	Comorbidities	Procedural aspects
Increasing age Female Sex Low body surface area Use of prior antiplatelet/antithrombotic agents	CKD Diabetes Liver Disease Anaemia Prior Bleeding Previous cardiovascular interventions	CPB Time Number of bypass grafts IABP

CKD – Chronic Kidney Disease; CPB - Cardiopulmonary bypass time; IABP – Intra aortic balloon pump

Analysing how these factors affect the bleeding risk individually and as a combination is often challenging. Hence, risk stratification tools can be used to aid the decision-making process (Table 2). The PRECISE-DAPT score, originally developed to predict out-of-hospital bleeding risk in patients undergoing coronary stenting [26] has also been evaluated in CABG populations. In a large Swedish observational study with over 22,000 CABG patients [27], PRECISE-DAPT demonstrated only moderate discriminatory accuracy and consistently underestimated the bleeding risk in patients treated with DAPT.

**Table 2:** Summary of bleeding risk scores, studies, and definitions in patients undergoing coronary artery bypass grafting (CABG)

Study / Tool	Study Design	Population	Bleeding Definition / Outcome	Clinical Utility
PRECISE-DAPT score [27]	Risk prediction model	PCI	Predicts out-of-hospital bleeding	Limited utility in CABG; not calibrated for surgical populations
ARC-HBR criteria [28]	Consensus definition	PCI	Defines high bleeding risk using major/minor criteria	Not applicable to CABG
Papworth score [29]	Risk prediction model	CABG	Predicts perioperative bleeding risk using clinical and procedural variables	Limited clinical uptake
TRUST score [30]	Risk prediction model	CABG	Estimates risk of bleeding and transfusion following cardiac surgery	Limited clinical uptake
WILL-BLEED score [31]	Risk prediction model	CABG	Incorporates variables such as anaemia, female sex, renal dysfunction	Promising but not routinely used; requires further validation
European multicentre CABG study [32]	Observational study	CABG	Standardised reporting of bleeding outcomes	Primarily research use
Universal Definition of Perioperative Bleeding (UDPB) [33]	Consensus definition	CABG	Classifies bleeding severity based on postoperative variables	Primarily research use

PCI – Primary Coronary Intervention; CABG – Coronary Artery Bypass Grafting

The Academic Research Consortium High Bleeding Risk (ARC-HBR) criteria define patients undergoing PCI as high bleeding risk if they meet specific criteria [28]. However, this classification is not directly applicable to CABG patients, as it was derived and validated in PCI populations receiving dual antiplatelet therapy, and does not account for the distinct perioperative bleeding risks and surgical factors specific to cardiac surgery.

There are however other risk scoring systems specifically for patients undergoing CABG including, Papworth [29] and TRUST [30]. Among these, the WILL-BLEED score has demonstrated superior discriminatory ability [31]. Variables involved in the risk model include preoperative anaemia, female gender, eGFR < 45 ml/min/1.73 m^2^, potent antiplatelet drugs discontinued less than five days, critical preoperative state, ACS and perioperative anticoagulant use.

The European multicentre study on CABG [32] and Universal definition of perioperative bleeding UDPB classifications [33] standardise the reporting of perioperative bleeding after cardiac surgery using postoperative variables such as transfusion requirements and re-exploration. However, as they are applied retrospectively and do not inform real-time clinical decision-making, they have limited utility in routine CABG practice and are rarely used outside research settings. Ultimately, none of these scores are used routinely in clinical practice. Currently available models have very modest sensitivity and specificity and are derived from single-centre cohorts or selected populations. Hence, optimal bleeding risk assessment requires integration of procedural and patient factors and clinician judgment, with risk scores serving as an adjunct.

## Platelet function tests

Platelets play a central role in haemostasis by rapidly forming a plug at sites of vascular injury. Impaired platelet function increases bleeding risk, whereas increased platelet reactivity is associated with increased thrombotic risk. Platelet function testing, such as aggregometry and flow cytometry, are performed in laboratories, while point-of-care systems allow bedside testing to evaluate platelet disorders and monitor antiplatelet therapy in real-time clinical settings [34].

Preoperative platelet function testing may have a role in selected patients to guide the timing of cardiac surgery in those receiving antiplatelet therapy. Current guideline-recommended washout periods do not account for individual variability in platelet recovery [35]. Observational studies suggest that measuring platelet function preoperatively can help identify patients with low or high platelet reactivity, allowing for a patient-centred approach to timing of surgery and potentially reducing bleeding complications [36, 37].

In contrast, the role of platelet function testing in the post-operative setting remains uncertain. Point of care platelet function tests show variable ability to predict blood loss and transfusion requirements in cardiac surgery [38]. In some settings their use was associated with significant reductions in blood loss and blood product transfusion [38]. These findings should be interpreted with caution. In patients undergoing CABG, cardiopulmonary bypass induces both platelet activation and dysfunction, and postoperative bleeding is inherently multifactorial; consequently, platelet function testing alone has limited positive predictive value for severe bleeding [39].

With respect to guiding antiplatelet therapy, randomised trials in PCI populations have demonstrated that platelet function–guided strategies can reduce ischaemic and bleeding events in the short term [40, 41]. However, in the context of cardiac surgery, platelet function testing cannot reliably distinguish drug-related platelet inhibition from surgery-induced platelet dysfunction, and no current guideline supports its routine use to guide postoperative antiplatelet therapy. Nevertheless, in highly selected, non-routine clinical scenarios, it may be considered as an adjunct to clinical judgement on an individualised basis.

## Genotype testing

Metabolism of clopidogrel is dependent on CYP2C19 enzyme activity. The gene encoding the CYP2C19 enzyme is highly polymorphic, resulting in some individuals having loss-of-function alleles and therefore reduced or no CYP2C19 enzyme activity (~ 30% of the population) [42]. Hence, these patients are poor metabolisers and have diminished clopidogrel responsiveness [43].

Evidence for genotype-guided antiplatelet therapy is derived almost exclusively from PCI populations, with limited data available in patients undergoing CABG. Several randomised trials have evaluated genotype-guided strategies in the PCI setting. In the POPULAR Genetics trial, a CYP2C19 genotype-guided strategy in STEMI patients undergoing primary PCI was non-inferior to routine ticagrelor or prasugrel for thrombotic outcomes and significantly reduced bleeding [44]. In the TAILOR PCI trial patients undergoing PCI who carried CYP2C19 loss-of-function variants, a genotype-guided P2Y12 inhibitor strategy did not significantly reduce ischaemic events compared with conventional clopidogrel therapy at 12 months [42]. Therefore, whilst in theory patients who have a defective gene may be able to be switched to prasugrel or ticagrelor (no genetic polymorphism), these findings indicate that the clinical benefit of genotype-guided antiplatelet escalation remains modest, inconsistent and its applicability to CABG populations is uncertain.

Importantly, the available evidence is almost exclusively derived from PCI populations, with a paucity of data to support its application in patients undergoing CABG. In practice, genotype-guided therapy after CABG should not be considered routine; however, in highly selected patients in whom clopidogrel remains the only feasible P2Y12 inhibitor despite suspected poor responsiveness (such as those with recurrent graft failure or recurrent ischaemic events despite adherence) CYP2C19 testing may be considered on an individualised, hypothesis-generating basis.

## Current guidelines (European Society of Cardiology (ESC)/ European Association for Cardio-Thoracic Surgery (EACTS)/ American Heart Association (AHA))

### Chronic Coronary Syndromes (CCS)

In patients undergoing CABG for CCS, lifelong aspirin therapy is pivotal in antiplatelet management with both the European and American guidelines consistently recommending early initiation of aspirin after surgery to reduce graft occlusion and ischaemic events.

The American Heart Association (AHA) recommends starting aspirin (100–325 mg daily) within 6 h postoperatively and continuing it indefinitely to prevent SVG occlusion and adverse cardiovascular outcomes (Class I, Level A) [15]. Similarly, the 2024 European Society of Cardiology (ESC) guideline for the management of CCS recommends lifelong aspirin at a dose of 75–100 mg daily (Class I, Level A) [45]. The 2024 EACTS guideline further emphasises the importance of early treatment, recommending that low-dose aspirin be (re)started within 24 h after surgery, ideally within 6 h, to reduce ischaemic events and graft failure (Class I, Level B) [1]. Despite minor differences in dosages across guidelines, the general clinical practice is to administer a 300 mg loading dose postoperatively, followed by a lifelong maintenance dose of 75 mg daily.

Dual antiplatelet therapy (DAPT) after CABG in CCS patients is not routinely recommended but may be considered in selected individuals. According to the AHA, a one-year course of DAPT with aspirin plus ticagrelor or clopidogrel may improve vein graft patency compared with aspirin alone [15]. Consistently, the ESC/EACTS guidelines state that DAPT may be considered in patients at higher risk of graft occlusion and at low risk of bleeding (Class IIb, Level B) [1, 45].

### Acute Coronary Syndromes (ACS)

In patients undergoing CABG in the context of an ACS, resumption of dual antiplatelet therapy after surgery is strongly recommended in the current guideline, reflecting the higher ischaemic risk in this population.

The 2023 ESC guidelines for the management of ACS recommend that patients who interrupt DAPT to undergo CABG should resume DAPT postoperatively for at least 12 months (Class I, Level C) [46]. Similarly, the 2024 ESC/EACTS guideline recommends that in patients with recent ACS or prior PCI, DAPT should be resumed or initiated after CABG as soon as it is considered safe, in order to reduce the risk of stent thrombosis and major cardiovascular events (Class I, Level B) [1].

However, it is important to recognise that this position has historically been supported largely by evidence derived from CABG subgroups within predominantly PCI/ACS studies rather than from dedicated CABG randomised trials.

## Antiplatelet monotherapy

### Aspirin Monotherapy

Aspirin has been the antithrombotic agent of choice for patients with CAD for over three decades (Table 3). Large meta-analyses incorporating randomised controlled trials have consistently demonstrated that aspirin reduces cardiovascular mortality and reduction of ischaemic events [47, 48]. Single antiplatelet therapy (SAPT) with aspirin is the current standard of care for most patients following CABG, particularly those with CCS. Low-dose aspirin (75–100 mg once daily) is recommended based on extensive evidence demonstrating reductions in cardiovascular morbidity and mortality, including in patients with prior MI [47, 49].

**Table 3:** Summary of key studies evaluating aspirin monotherapy after CABG

Study	Design	Population (*n*)	Intervention vs. Comparator	Primary Outcomes
**Lorenz et al. (1984)** [50]	RCT	CABG (*n* = 200)	Aspirin vs. placebo	Improved graft patency
**Fremes et al. (1993)** [51]	Meta-analysis	CABG (*n* = 1,443)	Aspirin vs. placebo	Decreased SVG occlusion (OR 0.60, 95% CI 0.51–0.71; *P* < 0.0001)
**Mangano et al. (2002)** [4]	Observational cohort study	CABG (*n* = 5,065)	Aspirin vs. placebo	Decreased mortality after CABG
**Antithrombotic Trialists’ Collaboration (2002**,** 2009)** [47, 48]	Meta-analysis	High-risk vascular patients (*n* > 100,000)	Aspirin vs. placebo	Decreased cardiovascular mortality and ischaemic events
**Björklund et al. (2020)** [49]	Registry-based cohort study (SWEDEHEART)	CABG (*n* = 28,000)	Aspirin vs. placebo	Decreased long-term mortality

RCT- Randomised Controlled Trial; CABG – Coronary Artery Bypass Grafting; SVG Saphenous Vein Graft

Early placebo-controlled randomised controlled trials established aspirin’s role in preventing SVG occlusion after CABG [50]. A meta-analysis of 17 RCTs involving 1,443 patients showed that aspirin significantly reduced graft occlusion compared with placebo (OR 0.60, 95% CI 0.51–0.71; *P* < 0.0001) [51]. Initiation of low to medium-dose aspirin (100–325 mg daily) within 6 h of surgery was associated with improved graft patency without an excess risk of bleeding [52]. Aspirin has also been associated with reduced mortality and ischaemic complications following CABG [4].

### P2Y12 inhibitor monotherapy

Current evidence does not support P2Y12 inhibitor monotherapy as an alternative to aspirin after CABG. Trials evaluating clopidogrel or ticagrelor monotherapy have failed to demonstrate superiority over aspirin for SVG patency [53, 54]. An individual patient data meta-analysis showed no significant reduction in graft failure with ticagrelor monotherapy compared with aspirin, although the point estimate favoured ticagrelor (OR 0.86, 95% CI 0.58–1.27) [55].

## Dual antiplatelet therapy (DAPT)

Dual antiplatelet therapy, consisting of aspirin plus a P2Y12 inhibitor, is well established in patients with ACS and after PCI where it reduces thrombotic complications and adverse cardiovascular events compared with aspirin alone [56]. However, DAPT is associated with an increased risk of major spontaneous and surgical bleeding [13].

## Evidence for DAPT after CABG

Several studies suggest that DAPT may reduce graft failure and adverse clinical outcomes after CABG, particularly in patients presenting with ACS. Observational and subgroup analysis from ACS trials have demonstrated reduced graft failure, all-cause mortality, and ischaemic events in CABG patients treated with DAPT following ACS [57, 58]. This benefit appears more consistent with ticagrelor-based DAPT than with clopidogrel, while evidence in patients with CCS remains limited and less convincing [55]. However, these studies were not specifically designed to determine the optimal postoperative antiplatelet regimen after CABG, and their findings should therefore be interpreted as hypothesis-generating rather than definitive.

More recent randomised evidence has challenged the assumption that improved graft patency translates into early clinical benefit. In the TACSI trial, Jeppsson et al. randomised 2,201 patients undergoing CABG for ACS to receive ticagrelor plus aspirin or aspirin alone for one year [59]. Ticagrelor-based DAPT did not reduce the incidence of the composite endpoint of death, myocardial infarction, stroke, or repeat revascularisation compared with aspirin monotherapy, but was associated with a significantly higher risk of major bleeding and net adverse clinical events [59].

These findings are consistent with prior meta-analysis data. An individual patient data meta-analysis of four randomised trials including 1,316 patients and 1,668 vein grafts demonstrated that ticagrelor-based DAPT significantly reduced vein graft failure compared with aspirin alone (11.2% vs. 20.0%), but at the cost of a markedly increased risk of Bleeding Academic Research Consortium (BARC) type 2, 3, or 5 bleeding events (22.1% vs. 8.7%) [55]. Taken together, the available evidence suggests that while DAPT, particularly with ticagrelor, may improve graft patency, this does not consistently translate into short-term clinical benefit and is offset by excess bleeding.

## Duration and de-escalation of DAPT

Given the competing risks of graft failure and bleeding after CABG, recent studies have explored shorter or de-escalated DAPT strategies. The TOP-CABG trial [60] directly addressed this question by comparing standard 12 months of DAPT with a de-escalated strategy consisting of 3 months of DAPT followed by 9 months of aspirin monotherapy. De-escalated therapy was non-inferior to standard DAPT with respect to graft occlusion (graft occlusion occurred in 10.79% of grafts in the de-escalated DAPT group and 11.19% in the standard DAPT group (difference − 0.31%; 95% CI − 3.13 to 2.52; *p* = 0.008 for noninferiority), while significantly reducing clinically relevant bleeding events (DAPT (8.26% vs. 13.19%; HR 0.62; 95% CI 0.48–0.81; *p* < 0.001).

Longer-term data, however, suggest that the timing and population context of DAPT may be critical. In the DACAB trial, conducted in China, 500 patients undergoing CABG (approximately two-thirds presenting with ACS) were randomised to ticagrelor plus aspirin, ticagrelor alone, or aspirin alone for 12 months. Although no significant differences in clinical outcomes were observed at one year, extended 5-year follow-up demonstrated a lower incidence of major adverse cardiovascular events among patients initially treated with DAPT compared with aspirin monotherapy [61]. This apparent long-term benefit contrasts with the neutral one-year clinical results of the TACSI trial reported by Jeppsson et al. [59]. However, the DACAB trial was smaller, conducted in a single country, and included routine angiographic assessment of graft patency, supporting the hypothesis that early improvements in graft patency may translate into delayed clinical benefit. In contrast, TACSI was a large, pragmatic, registry-based trial conducted across multiple Nordic countries, with high uptake of guideline-directed medical therapy, including more intensive secondary prevention such as high-intensity statins, and low overall event rates, potentially attenuating any incremental benefit of prolonged DAPT. More broadly, differences in adherence to secondary prevention measures, including lipid-lowering therapy, blood pressure control, and other components of guideline-directed care, may have influenced long-term outcomes and further limit direct comparability between trials. Differences in patient selection, background bleeding risk, adherence to ticagrelor, and duration of follow-up further limit direct comparability between studies. Importantly, graft patency was not assessed in TACSI, precluding mechanistic linkage between platelet inhibition and long-term outcomes.

The term “de-escalation” may refer not only to shortening the duration of DAPT but also to reducing P2Y12 inhibitor potency, for example by switching from ticagrelor or prasugrel to clopidogrel after an initial high-risk period. In PCI and ACS populations, guided or unguided potency-based de-escalation strategies have been shown to reduce bleeding without a clear loss of ischaemic protection in selected patients [41, 44]. Although no dedicated trials have evaluated this approach after CABG, the concept may be cautiously extrapolated to carefully selected postoperative patients in whom ongoing DAPT is indicated (e.g., recent ACS or prior PCI), but bleeding risk increases after the early postoperative phase.

Taken together, these data suggest that any potential long-term benefit of prolonged DAPT may be confined to selected populations and remains uncertain in the absence of consistent early clinical benefit, whereas shorter or de-escalated strategies may provide a more favourable balance between efficacy and safety for many patients.

## Combination therapy with oral anticoagulants (OAC)

In patients requiring oral anticoagulation (e.g., those with non-valvular atrial fibrillation (AF), venous thromboembolism (DVT/PE), or mechanical heart valves), combination therapy strategies have been explored.

In a sub-study of the COMPASS trial, the factor Xa inhibitor rivaroxaban, either alone or in combination with aspirin, did not reduce one-year graft failure compared with aspirin monotherapy [62]. These findings do not support the routine use of oral anticoagulant-based strategies for graft protection after CABG in the absence of other indications, while recognising that selected patients, such as those with high polyvascular risk, may still derive benefit from systemic vascular protection strategies beyond graft patency.

For patients with a prior indication for oral anticoagulation, resumption of OAC in combination with SAPT, such as aspirin or clopidogrel, should occur as early as clinically feasible postoperatively. Triple antithrombotic therapy should be avoided in these patients due to the elevated risk of bleeding. After 12 months post-CABG, it is reasonable to discontinue the antiplatelet agent and continue OAC alone, in line with current recommendations for patients with AF undergoing PCI [63].

## Conduit type and surgical strategy

The clinical relevance of graft patency after CABG is strongly influenced by conduit type and time since surgery, and this distinction is essential when interpreting the antiplatelet literature. Much of the rationale for intensified antiplatelet therapy after CABG, including the potential benefit of DAPT, relates primarily to prevention of early SVG thrombosis. SVGs are more susceptible than arterial conduits to early platelet-mediated thrombotic occlusion and later atherosclerotic degeneration, making them the principal substrate in which enhanced platelet inhibition may plausibly improve graft patency [53, 54]. Accordingly, most CABG-specific evidence suggesting improved graft patency with DAPT has been driven predominantly by SVG outcomes rather than arterial grafts.

By contrast, arterial grafts, particularly the LIMA, have superior long-term patency and distinct mechanisms of failure, including technical factors, competitive flow, and spasm, rather than the same degree of platelet-driven thrombotic vulnerability seen in SVGs. As a result, the biological rationale for routine DAPT to improve arterial graft patency is less compelling, and current evidence does not support assuming that arterial conduits derive the same benefit from intensified antiplatelet therapy as SVGs.

This distinction has practical implications for surgical strategy. In patients receiving CABG with one or more SVGs, especially in the setting of ACS or other features suggesting high thrombotic risk, the potential patency benefit of DAPT may be more relevant, although this must still be balanced against bleeding risk and the absence of consistent early clinical outcome benefit in contemporary trials. Conversely, in patients undergoing multiple arterial grafting, the incremental rationale for routine DAPT is likely attenuated, and aspirin monotherapy may be sufficient in the absence of another compelling indication for intensified platelet inhibition. Therefore, conduit selection and overall grafting strategy should be considered explicitly when moving towards patient-centred antiplatelet therapy after CABG.

## Conclusions

Patient-centred antiplatelet therapy after CABG requires a fundamentally different approach from PCI, with treatment decisions driven primarily by surgical bleeding risk, graft type, and clinical presentation rather than extrapolated PCI-based strategies. Aspirin monotherapy remains the cornerstone of antiplatelet therapy following CABG. Dual antiplatelet therapy may confer additional benefits in carefully selected high-risk patients, particularly those presenting with ACS and those who had SVG grafts. This potential advantage must be weighed against a higher risk of bleeding and the absence of consistent improvement in early clinical outcomes in contemporary CABG-specific trials. Emerging evidence suggests that shorter or de-escalated DAPT strategies may offer a more favourable balance between efficacy and safety. Taken together, the available evidence supports a move away from “one-size-fits-all” antiplatelet regimens after CABG toward a patient-centred approach. Future research should prioritise refinement of clinical risk stratification and may include the role of platelet function testing or genotype-guided strategies in highly selected patients.

## Data Availability

No datasets were generated or analysed during the current study.
